# Pelvic Arterial Embolisation in Obstetric and Gynaecological Haemorrhage: A Single-Centre Case Series

**DOI:** 10.1155/ogi/9932410

**Published:** 2025-08-17

**Authors:** Monica Narula, Tuan Phan, Kiran Atmuri

**Affiliations:** ^1^Mercy Hospital for Women, Heidelberg, Victoria, Australia; ^2^Peninsula Health, Frankston, Victoria, Australia

**Keywords:** artery embolisation, gynaecology, haemorrhage, interventional radiology, obstetrics

## Abstract

**Background:** Obstetric and gynaecological haemorrhage contributes significantly to morbidity and mortality globally. Pelvic arterial embolisation has been described as a management option for emergency haemorrhage not responsive to conservative measures. Despite encouraging outcomes, it is not widely utilised.

**Aim:** This study aims to assess efficacy and early complications of pelvic arterial embolisation as a therapy for obstetric and gynaecological haemorrhage not controlled by conservative methods.

**Materials and Methods:** This retrospective single-centre case series reviewed all cases of acute haemorrhage from obstetric or gynaecological causes referred for angiographic embolisation between 2014 and 2020.

**Results:** Twelve patients underwent pelvic arterial embolisation with a 100% technical success rate and 91.6% clinical success rate. There were no major early complications.

**Conclusion:** Pelvic arterial embolisation is a safe and effective option for obstetric and gynaecological haemorrhage not responsive to conservative management.

## 1. Introduction

Haemorrhage in obstetrics and gynaecology contributes significantly to morbidity and mortality. The World Health Organization estimates that postpartum haemorrhage (PPH) contributes to 35% of maternal deaths worldwide, with the incidence in Australia ranging between 5% and 15% [1]. In gynaecological cases, postoperative bleeding and bleeding due to conditions such as genital tract malignancies can be life-threatening and mandate immediate management.

Following conservative measures to manage obstetric and gynaecological haemorrhage, patients often proceed to theatre for definitive surgical management. Surgical treatments expose patients to risks of anaesthesia and complications of further surgery, which may have greater consequences in older patients with medical comorbidities. In obstetric cases, stepwise devascularisation of the uterus, such as internal iliac or uterine artery ligation, has highly variable success rates due to collateral circulation and can be technically challenging resulting in further vascular injury [2]. There is also potential loss of reproduction if a salvage hysterectomy is performed.

Interventional radiology in the form of pelvic arterial embolisation (PAE) is minimally invasive and has historically been employed primarily in elective contexts, such as management of uterine leiomyomas and arteriovenous malformations (AVMs). Over recent decades, its application has expanded significantly into emergency contexts, notably obstetric and gynaecological haemorrhage. This evolving role positions PAE as an important procedure for avoiding surgery in women following childbirth, after gynaecological surgery, or in women presenting with intractable genital tract bleeding from malignancies.

Existing literature on the utility of PAE has a focus on acute obstetric haemorrhage, predominantly exploring the use of internal iliac and uterine artery embolisation in the perioperative management of placental adhesive disorders and severe PPH [3, 4]. High rates of success have been consistently reported, ranging between 88% and 100%, [4–6]. Together with an acceptable profile of complications [3, 7] and subsequent fertility rates of 70%–80% [8, 9], PAE presents a compelling option when compared with hysterectomy. Case reports also describe nonemergent embolisation of uterine artery pseudoaneurysms during pregnancy, with positive outcomes [10, 11].

In the gynaecology setting, PAE is utilised primarily in elective leiomyoma and arteriovascular malformation cases, which have been widely accepted and reported on [12–14]. Its role in controlling emergency gynaecological haemorrhage has had less attention [2]. Several small studies cite the use of embolisation in intractable postoperative and posttermination haemorrhage [15–17]. Three recent case series demonstrated high technical success rates (95.2%, 95.7% and 100%) for patients with inoperable gynaecological malignancies, with few complications [18–20]. Large studies and meta-analyses assessing PAE in emergency gynaecology are lacking.

The aim of this study is to assess the safety and efficacy of PAE in both obstetric and gynaecological haemorrhage. In clearly delineating the indications, clinical efficacy and complications associated with PAE in these distinct contexts, this study aims to contribute to international literature on the evolving role of PAE. To the investigators' knowledge, no comparable study evaluating both obstetric and gynaecological emergency use of PAE has previously been published in the Australian context.

## 2. Materials and Methods

### 2.1. Study Setting

The study was based at a public hospital located in outer metropolitan Melbourne in Australia. The study site is a second-level trauma centre and has a maternity unit where women birth approximately 3000 babies each year.

### 2.2. Ethics

The study has received ethics approval from the Peninsula Health Human Research Ethics Committee (reference number: QA/65013/PH-2020-215637(v1)).

### 2.3. Data Collection

The hospital electronic radiology database was used to identify female patients who had clinically suspected haemorrhage from an obstetric or gynaecological cause requiring PAE between 2014 and 2020, inclusive. Patients who received PAE in elective admissions were excluded from the study. Patient demographic, procedure technical details and clinical history were obtained from the hospital electronic patient health record and radiology database.

### 2.4. Embolisation Technique

All patients provided written informed consent before embolisation procedures. Embolisation procedures were performed by one of five interventional radiologists, with experience ranging from 2 to 9 years. Each procedure varied slightly depending on location of bleeding, but the standardised steps involved in the procedure are herewith described. A 5-French introducer sheath (Cordis, Miami Lakes, Florida, USA) was inserted into the right common femoral artery. Nonselective pelvic angiograms (Artis Zee, Siemens Medical Solutions, Erlangen, Germany) were performed from the level of the lower abdominal aorta using a pigtail catheter (Boston Scientific, Boston, MA, USA). Both internal iliac and uterine arteries were selectively catheterised using a 5-French C2 catheter (Cook Medical, Bloomington, IN, USA) and angiography subsequently performed. Embolisation was performed in the presence of active bleeding (contrast extravasation), pseudoaneurysm, arteriovenous fistula, neovascularity, arterial spasm or abrupt cutoff. Superselective catheterisation of the bleeding artery was performed using a Progreat microcatheter (Terumo, Tokyo, Japan). Embolisation was performed using microcoils (Cook Medical, Bloomington, IN, USA), Gelfoam (Pfizer, New York, USA) or polyvinyl alcohol (PVA) particles in the range of 500–710 microns (Boston Scientific, Boston, MA, USA).

### 2.5. Definitions

Technical and clinical success rates were measured against interventional radiology standards of practice [21]. Technical success is defined as successful catheterisation and embolisation of the target artery with the absence of radiological signs of continued bleeding, such as contrast extravasation. Clinical success is defined as sustained haemostasis after embolisation without rebleeding within 30 days.

There is no consensus definition of haemodynamic instability. In this study, haemodynamic instability was defined as clinical features of circulatory shock where there was a systolic blood pressure lower than 90 mm Hg or a heart rate greater than 120 beats per min despite adequate medical management, or in cases requiring continuous vasopressor support [21]. Nonovert disseminated intravascular coagulopathy (DIC) was diagnosed based on criteria established by the International Society on Thrombosis and Haemostasis [22].

Complications were classified as major or minor and early or late. Major complications were defined as those requiring further intervention, an unplanned increase in care requirements (e.g., infection or procedural trauma), permanent sequelae (e.g., uterine necrosis, ischaemia, premature ovarian failure or Asherman syndrome) or death. Minor complications were defined as self-limiting conditions that required no specific therapy, such as transient pain, fever or postembolisation syndrome.

Early complications are defined as occurring in the immediate postprocedure window (typically within 48–72 h after the procedure). These commonly include puncture site haematoma, transient pelvic pain or nausea. Late complications were defined as occurring after initial full recovery from the procedure, typically beyond the immediate postprocedural period. Late complications may include reproductive sequelae such as fertility impairment or chronic pelvic pain [8, 23].

## 3. Results

Twelve cases were identified with the mean age of 43.92 years. Four cases (33%) were due to obstetric reasons and 8 (66%) cases due to gynaecological reasons. Of the obstetric causes, two resulted from vaginal trauma following birth and two due to uterine rupture. Five of the 8 gynaecological cases were in the setting of postoperative bleeding. The index surgeries included hysterectomies of various subtypes and a myomectomy. The remaining 3 gynaecological cases were due to presentations to the emergency department with vaginal bleeding from a fibroid, AVM and cervical cancer. The most common route of bleeding was vaginal (*N* = 7) and the remaining had intraperitoneal haemorrhage (*N* = 5).

Patient characteristics, haemodynamic status and management prior to undergoing PAE are summarised in Table 1. Pre-embolisation care included localised repair of vaginal tears, uterotonics following birth, transfusion of blood products, fluid resuscitation and vaginal packing. Eleven of the 12 patients required blood products with the packed red cell unit requirement ranging from 1 to 27 (mean 7.3) units. Blood loss varied between 1 and 8.5 L. Four patients were haemodynamically unstable immediately prior to embolisation. All four of these patients were already intubated pre-embolisation with three patients (Cases 2, 9 and 11) being transferred to the interventional radiology suite directly from the operating table and one patient (Case 6) transferred from intensive care. One patient (Case 2) had evidence of nonovert DIC. The time taken between decision for embolisation and procedure start time ranged from 30 min to 24 h. All decisions made intraoperatively occurred within 60 min (Cases 2, 9 and 11). Two patients (Cases 9 and 12) were embolised outside the standard working hours of 7 a.m.–6 p.m. Cases 1 and 5 had a longer interval between decision to embolise and procedure completion; they were afterhours emergency presentations and after initial assessment and conservative management, the bleeding had reduced and so embolisation was performed the following morning.

Embolisation findings are summarised in Table 2. Technical success was achieved in all 12 patients (100%) and clinical success in 11 patients (91.6%). One patient (Case 11) required re-embolisation due to clinical failure (rebleeding) in the context of cervical cancer and abnormal neovascularity of the left uterine artery which had been embolised previously.

**Figure 1:**
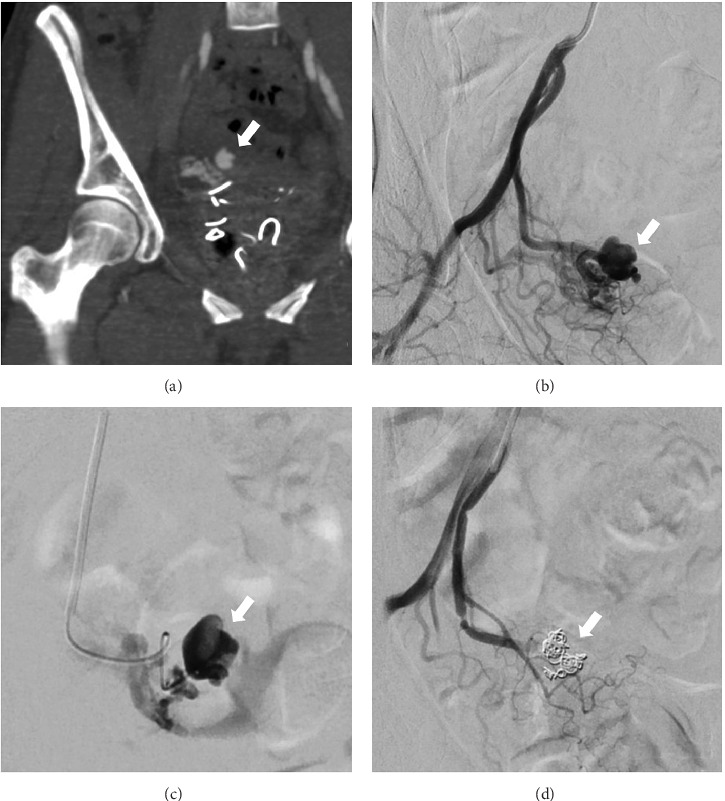
Case 1. (a) CT angiogram showing 10 mm pseudoaneurysm (arrow) arising from the distal right uterine artery. Angiograms show (b) selective catheterisation of the anterior branch of the internal iliac artery and a pseudoaneurysm arising from the right uterine artery (arrow), (c) superselective catheterisation of the right uterine artery and the pseudoaneurysm with associated small arteriovenous fistula (AVF), as seen by the early venous filling, and (d) successful occlusion of the pseudoaneurysm and AVF with coils (arrow).

**Figure 2:**
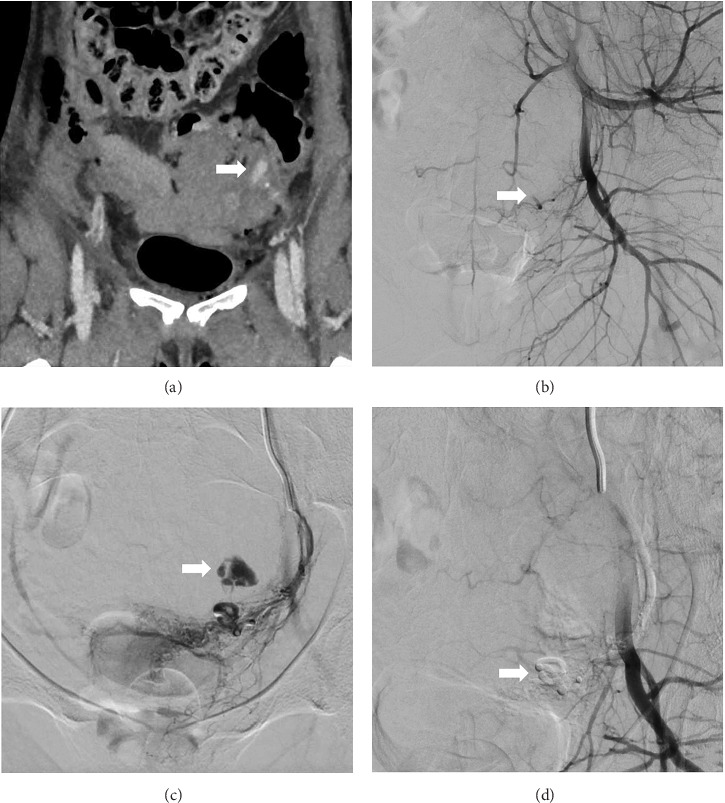
Case 8. (a) CT angiogram after bleeding from abdominal hysterectomy showing a left pelvic haematoma with active contrast extravasation, consistent with site of active bleeding (arrow). Angiograms show (b) selective catheterisation of the left internal iliac artery showing abrupt termination of the left uterine artery from vasospasm (arrow), (c) superselective catheterisation showing active bleeding (arrow) and (d) successful haemostasis following embolisation with Gelfoam (arrow).

Various vessels were embolised including unilateral or single uterine arteries (*N* = 7), internal iliac artery embolisation (*N* = 2), vaginal artery embolisation (*N* = 2) and inferior epigastric arterial embolisation (*N* = 1). Angiography findings showed active bleeding in 6 patients and abnormal vascularity in 4 patients (2 of which exhibited pseudoaneurysms), and 3 patients had no significant abnormalities. Figures 1 and 2 show examples of CT and angiographic findings with pre- and postembolisation images. The most commonly used embolic agent was Gelfoam (*N* = 6, 50%), followed by coils (*N* = 3, 25%), PVA particles (*N* = 2, 16.7%) and combined Gelfoam with coils (*N* = 1, 8.3%).

One patient (Case 3) had postembolisation syndrome, exhibiting pain, nausea and loss of appetite, which was managed conservatively. There were no other documented major or minor complications of the embolisation procedure. One patient (Case 6) had deceased 3 weeks after embolisation due to advanced stage ovarian cancer. None of the cases required hysterectomy to control haemorrhage.

## 4. Discussion

The present study shows that PAE is an effective procedure in the management of acute obstetric and gynaecological haemorrhage not amenable to conservative measures, with all 12 patients successfully undergoing embolisation. These findings are in line with existing literature, where success rates in comparable studies range from 88% to 100% [5, 6, 15]. In studies looking primarily at PPH, success rates are approximately 90%–99% [3, 4, 7, 23, 24].

### 4.1. Role of PAE in Obstetric Haemorrhage

PAE offers a nonsurgical and uterine-sparing option for women. None of the patients in the study required surgery, and hysterectomy was successfully avoided in all women with an intact uterus at the time of embolisation. This is particularly important since most obstetric patients undergoing emergency PAE are of reproductive age, and uterine preservation is crucial for maintaining fertility.

Aside from PPH arising from uterine atony or placental disorders, our study demonstrated that PAE can also be highly effective in complex obstetric scenarios such as vaginal trauma and uterine rupture, where direct surgical repair can be technically challenging due to anatomical access difficulties and tissue fragility. These findings further show the versatility and clinical utility of embolisation in obstetric emergencies, consistent with prior literature [4, 5, 21].

### 4.2. Role of PAE in Gynaecological Haemorrhage

In the gynaecological setting, PAE plays a similarly critical role but is often employed under different clinical circumstances. Gynaecological haemorrhage typically involves postoperative bleeding or genital tract bleeding due to malignancy. Surgical management in these scenarios is frequently challenging due to anatomical complexity and the generally poor clinical status of older patients with medical comorbidities [18].

In our study, five out of eight gynaecological cases presented with postoperative haemorrhage following procedures such as hysterectomy and myomectomy, showing the role of embolisation as an effective intervention in managing severe bleeding in surgical contexts. The remaining cases involved acute bleeding from gynaecological malignancies and vascular malformations, further illustrating the broad applicability of PAE. These findings are consistent with recent case series reporting similarly high success rates in gynaecological malignancy–related haemorrhage (95.2%–100%), often in patients unsuitable for further surgical interventions [18–20, 25].

Our findings show a particular clinical scenario where a patient with cervical cancer required re-embolisation due to abnormal tumour-associated neovascularity. Embolisation in malignancy cases is often palliative, aiming primarily to reduce morbidity and repeated surgical interventions such as recurrent vaginal packing [6, 18, 19]. Such patients, frequently elderly and medically compromised, derive significant clinical benefit from this minimally invasive approach, as major surgical interventions carry a substantially higher perioperative risk.

PAE also offers advantages over surgical interventions even when the source of bleeding is not evident. The clinical success of embolisation is not limited to angiographic evidence of active haemorrhage at the time of examination. In 3 cases in our study, there was no angiographic evidence of active haemorrhage. This can be explained by intermittent or temporary cessation of haemorrhage caused by arterial vasospasm. If there is clinical evidence of haemorrhage, but still absence of angiographic evidence, proceeding with embolisation using a temporary haemostatic agent such as Gelfoam is useful in preventing further morbidity.

### 4.3. Complications of PAE

The patients embolised in our study experienced no major early complications, and only one patient experienced postembolisation syndrome. Postembolisation syndrome, defined as transient pelvic pain, low-grade fever, nausea and loss of appetite typically occurring within 48–72 h after embolisation, arises due to tissue ischaemia and necrosis [5]. It is a self-limiting condition and represents the most common minor complication of PAE. However, it can prolong hospital admission duration. Overall, our study aligns with expected rates of minor complications, with a systematic review citing a 13% risk of postembolisation syndrome [21].

Serious major complications of PAE, although rare, must be clearly acknowledged and discussed given their potential long-term clinical significance, particularly, in the younger patient. Uterine necrosis is a rare but serious sequela of PAE for PPH [9, 26]. Two small case series of 11 patients each found uterine necrosis rates of 7.97% and 9.4% [23, 26]. Larger studies citing incidence are unavailable. Time to presentation and symptoms of uterine necrosis are variable, but may include fever, pain and abnormal vaginal discharge. Uterine necrosis, if it occurs, may necessitate hysterectomy [27].

Reproductive outcomes following PAE are generally favourable, but specific complications and implications must be explicitly considered. A systematic review reported a return to normal menses in 81.3% of patients post-PAE, with Asherman syndrome (uterine synechiae) noted as a possible consequence with an incidence of approximately 2% [8]. Asherman syndrome may cause cyclical pain and abnormal uterine bleeding and lead to subfertility. Fertility outcomes following PAE have generally been reassuring, with subsequent pregnancy rates of approximately 70%–80%, consistent with baseline population fertility rates [8, 9, 28–30]. Premature ovarian failure or reduced ovarian reserve may result from nontarget embolisation, particularly when permanent embolic agents are used or when embolisation occurs in close proximity to ovarian artery anastomoses [31, 32]. A 2016 retrospective analysis investigating ovarian reserve following PAE for fibroids noted a significant reduction in anti-Mullerian hormone levels and antral follicle count 3 months after PAE [31]. These reductions showed recovery at 12 months in patients < 40 years of age, but not in patients > 40 years. The hypothesised mechanism is embolic agent effect on ovarian circulation via anastomoses, and younger women have a greater potential for recovery [32]. However, several other studies prior to 2016 found no significant reduction in ovarian reserve, and therefore robust conclusions remain controversial [33]. Where pregnancy does occur, potential complications include increased risks of abnormal placentation (placenta accreta spectrum) and recurrent PPH with incidences of 16.3% and 19.2%–24%, respectively, reported in meta-analyses [9]. There are conflicting data on the risk of subsequent preterm birth and intrauterine growth restriction [9, 21, 34].

At a technical level, Gelfoam is preferred over permanent embolic particles in women wishing to preserve fertility, as it allows for vessel recanalisation within 10–14 days postprocedure, enabling future pregnancies [9]. Superselective embolisation techniques should be prioritised to minimise risks of ovarian or uterine complications.

Given the potential for significant long-term reproductive sequelae, establishing clear protocols for patient selection, informed consent and comprehensive follow-up is critical. Units should be encouraged to systematically document both short-term and long-term complication data to facilitate robust future analysis and reporting. Establishment of interhospital clinical registries may assist this process [8, 9, 26].

In the gynaecological literature, there is limited comment on late complications of embolisation in the acute setting. In three case series involving PAE for haemorrhage due to gynaecological malignancy, there were no major complications, and a low rate of minor complications that settled with conservative measures [18–20].

### 4.4. Further Considerations for PAE

This study highlights that haemodynamic instability should not be an absolute contraindication to PAE. Four of the 12 patients in our study were haemodynamically unstable immediately prior to embolisation, all requiring ionotropic support. These favourable findings are consistent with existing studies [4, 5, 21] where haemodynamic instability was not a contraindication to PAE. DIC is a concern for ongoing bleeding, procedure success rates and puncture site haematoma [4]. Coagulopathies should be corrected prior to embolisation as it is the least invasive method of slowing bleeding. Correction of coagulopathies is particularly important for retroperitoneal bleeding where there can be multiple sources of bleeding. This may be a time-limiting step; however, modern coagulation point-of-care testing which focusses on viscoelastic properties of blood may help facilitate early recognition and prompt correction of DIC.

Prompt transfer to the angiogram suite is required for successful outcomes in PAE for acute haemorrhage. One major barrier to PAE is lack of access to interventional radiology services. A collaborative effort is needed between obstetrician gynaecologists and interventional radiologists. Clear referral pathways, treatment protocols, trained interventional radiologists and early communication between teams are key to success of PAE in the acute setting. Three of our 12 cases were performed within 60 min due to early intraoperative decision for PAE. Our hospital has appropriately trained and motivated interventional radiologists who are on-call with ancillary support staff 24 h a day. Two cases were performed after-hours and one patient was an interhospital transfer. In practice, within working hours, there can be a 30–60 min delay between the decision to embolise and the embolisation being completed. This reflects the time required for transfer to the angiographic room, setting up equipment, gaining arterial access, catheterising the pelvic arteries, performing the angiogram and then embolising. After hours, a further 60 min may be needed to account for all radiology staff (interventional radiologist, radiographer and nurse) to be recalled to hospital.

### 4.5. Limitations of the Study

This study is not without limitations. The study's smaller sample size prevented multivariate statistical analysis that could be used to identify predictive factors for successful or failed PAE. Single-centre data may not be generalisable to all countries. The cost-effectiveness of PAE for acute haemorrhage was not studied. This may be particularly important in countries with limited financial resources. In hospitals where an existing on-call interventional radiology team exists, the financial burden may be less. The value of PAE must be balanced by the costs of inadvertent return to theatre and hysterectomies in women of reproductive age. Some of these costs may be difficult to quantify, such as the costs of infertility after hysterectomy and the impact on perceived quality of care with return to theatre for major surgery.

## 5. Conclusion

This study demonstrates that PAE offers high technical and clinical success rates, coupled with low early complication rates, in managing acute obstetric and gynaecological haemorrhage not responsive to conservative management. The distinct clinical contexts of obstetric and gynaecological haemorrhage have been explicitly addressed, highlighting PAE's versatility and effectiveness in both settings.

PAE provides a minimally invasive and uterine-sparing alternative to conventional surgical interventions, offering substantial clinical benefit, particularly when surgical options carry significant morbidity or fertility risks. Given its effectiveness and safety profile, obstetricians and gynaecologists should strongly consider PAE early in the acute management of severe haemorrhage, and multidisciplinary collaboration with interventional radiology services should be actively encouraged.

## Figures and Tables

**Table 1:** Patient characteristics, haemodynamic status and management prior to undergoing pelvic arterial embolisation.

Case	Age	Clinical history	Route of bleeding	EBL (mL)	Hb (g/L)	DIC	HI	Pre-embolisationmanagement	PRBC (units)	Time to embolisation
1	48	Post TAH, BS and unilateral oophorectomy	Vaginal	1800	109	−	−	Blood products, surgical exploration, packing	1	17 h
2	34	Vaginal trauma post instrumental birth	Vaginal	7000	48	+	+	Blood products, oxytocin, ergometrine, surgical repair, packing	12	35 min
3	41	Menorrhagia	Vaginal	#	66	−	−	Blood products, norethisterone, tranexamic acid	6	5.5 h
4	28	Uterine rupture, VBAC	Intraperitoneal	2500	51	−	−	Blood products, oxytocin, laparotomy	13	2 h
5	33	Menorrhagia	Vaginal	1000	94	−	−	Primolut	0	24 h
6	88	Post TAH and BSO	Vaginal	2000	66	−	+	Blood products, tranexamic acid, intubation and inotropic support	14	6 h
7	34	Post laparotomy for fibroid	Intraperitoneal	#	81	−	−	Blood products	3	3.5 h
8	34	Post sTAH and BS	Intraperitoneal	#	52	−	−	Blood products	3	2 h
9	36	Vaginal trauma post instrumental birth	Vaginal	8500	100	−	+	Blood products, surgical repair, tamponade, oxytocin, ergometrine, misoprostil, packing	27	30 min
10	49	Post sTAH and BS	Intraperitoneal	#	85	−	−	Blood products	3	40 min
11	53	Cervical cancer	Vaginal	3000	88	−	+	Blood products, tranexamic acid, surgical repair, packing	8	1 h
	53	Cervical cancer	Vaginal	1000	93	−	−	Blood products, packing	2	5 h
12	40	Uterine rupture, FDIU	Intraperitoneal	3000	89	−	−	Blood products, oxytocin, surgical repair	3	2.5 h

*Note:* TAH, total abdominal hysterectomy (sTAH = subtotal); Hb, haemoglobin (g/L); #, data not recorded.

Abbreviations: BS = bilateral salpingectomy, BSO = bilateral salpingo-oophorectomy, DIC = disseminated intravascular coagulopathy, EBL = estimated blood loss (mL), FDIU = foetal death in utero, HI = haemodynamic instability, PRBC = packed red blood cells, and VBAC = vaginal birth after caesarean.

**Table 2:** Angiographic data of pelvic arterial embolisation.

Case	Angiography finding	Embolised vessel	Embolic agent	Technical success	Clinical success	Duration (hr/m)	Complications/further management
1	Pseudoaneurysm and small AV fistula R UA, abnormal aberrant veins	R UA selective	Coils	+	+	1 h 45 m	Nil
2	Nil	Bilateral VA	Gelfoam	+	+	1 h 32 m	Nil
3	Fibroid vascularity	Bilateral UA	500–700 mic PVA particles	+	+	1 h 5 m	Postembolisation syndrome
4	R + L VA haemorrhage	Bilateral VA and R UA nonselective L VA superselective	Gelfoam	+	+	60 m	Nil
5	Abnormal vascularity, pseudoaneurysm left side of uterus	Bilateral UA	500–700 mic PVA particles	+	+	48 m	Nil
6	L IIA haemorrhage	L IIA superselective	Coils	+	+	1 h 47 m	Nil
7	Nil	Bilateral UA	Gelfoam	+	+	32 m	Nil
8	L UA haemorrhage	Bilateral UA	Gelfoam	+	+	24 m	Nil
9	L IIA haemorrhage	L IIA superselective	Coils	+	+	1 h 41 m	Nil
10	L IEA haemorrhage	L IEA	Microcoils	+	+	32 m	Nil
11	R UA haemorrhage	Bilateral UA	Coils, Gelfoam	+	—	1 h 27 m	Nil
	Abnormal neovascularity L UA	L UA	Gelfoam	+	+	1 h 30 m	Nil
12	Nil	Bilateral UA	Gelfoam	+	+	1 h 26 m	Nil

*Note:* AV, arteriovenous; L, left; R right; AVM, arteriovenous malformation.

Abbreviations: IEA = inferior epigastric artery, IIA = internal iliac artery, UA = uterine artery, and VA = vaginal artery.

## Data Availability

The data that support the findings of this study are available from the corresponding author upon reasonable request.
